# An Innovative AAL System Based on IoT Technologies for Patients with Sarcopenia

**DOI:** 10.3390/s19224951

**Published:** 2019-11-14

**Authors:** Filomena Addante, Federico Gaetani, Luigi Patrono, Daniele Sancarlo, Ilaria Sergi, Giuseppe Vergari

**Affiliations:** 1 Fondazione Casa Sollievo della Sofferenza, Department of Medical Sciences, Geriatrics Unit, 71013 San Giovanni Rotondo (FG), Italyd.sancarlo@operapadrepio.it (D.S.); 2 BionIT Labs s.r.l., 73100 Lecce, Italy; f.gaetani@bionitlabs.com; 3 Department of Engineering for Innovation, Università del Salento, 73100 Lecce, Italy; ilaria.sergi@unisalento.it (I.S.); giuseppe.vergari@studenti.unisalento.it (G.V.)

**Keywords:** Ambient Assisted Living, cloud, embedded systems, Internet of Things, mobile App, performance, sarcopenia, wearable device

## Abstract

Sarcopenia is a highly prevalent, age-related muscle disorder associated with adverse outcomes. It is very important from a medical point of view to periodically monitor patients at risk of developing sarcopenia in order to early detect its onset or progression through objective and specific indicators. Today, the emerging Internet of Things (IoT)-enabling technologies allow us to create innovative, wearable, and non-invasive systems that can offer useful clinical support in this area. This work is focused on the use of combined hardware and software technologies, enabling the IoT, in order to monitor people suffering from sarcopenia by offering a high value-added service in the field of the Ambient Assisted Living (AAL). In addition to the description of the proposed system architecture, a validation of the entire system is also included, from both a performance and a functional point of view. Test beds have been carried out by using the independent replications method, and all measurements related to the identified sarcopenia parameters are characterized by a 95% confidence interval with a 5% maximum relative error. The implementation of these technologies as a supporting clinical tool used in a specific setting could significantly impact the life and independence of the sarcopenic frail elderly population.

## 1. Introduction

Sarcopenia is a highly prevalent, age-related muscle disorder characterized by decreased muscle mass, decreased muscle strength and impaired physical performance. It is a process that starts with a slow rhythm at age 40–50 and grows after 60 years old. It causes muscle atrophy and compromises the quality of muscle tissue and it is responsible for symptoms such as a constant sense of weakness, poor balance, slow gait, and difficulty in performing basal activities of daily living (e.g., climbing stairs). Sarcopenia also is associated with an increased likelihood of adverse outcomes, including falls [1], fractures [2], physical disability [3], and higher mortality [4], and with enormous personal and societal costs [5,6]. Actually, sarcopenia has been overlooked and undertreated in mainstream practice because it is not so easy and feasible over the time to measure its three fundamental components—(i) muscle mass, (ii) muscle strength, and (iii) physical performance, such as gait speed [7].

A wide range of techniques can be used to assess these three parameters. Magnetic resonance imaging (MRI) and computed tomography (CT) are considered to be gold standards for non-invasive assessment of muscle mass [7]. However, these tools are not commonly used in primary care because of high equipment costs, lack of portability, and the requirement for highly trained personnel. Dual energy X-ray absorptiometry (DXA), a radiographic exam, is a more widely available instrument to determine muscle mass [8]. DXA is a radiographic exam able to determine

- bone density in different body parts, useful also during the diagnosis of osteoporosis;
- weight and percentage of lean mass and fat mass in different body areas that is fundamental to discover the stage of the disease.

The main drawback of DXA is that the equipment is not portable. A good portable alternative to DXA might be bioimpedance analysis (BIA), which is able to estimate the volume of fat mass and lean mass. However, it is often considered too inaccurate to provide reliable information. In reference [9], the authors propose an interesting critical review on available methods for muscle mass estimation in sarcopenia, highlighting strengths and weaknesses of each of them as well as their proposed field of application.

Relating to muscle strength, there are several techniques to measure this parameter. In particular, handgrip strength test is widely used to measure muscle strength [10]. Finally, in order to evaluate physical performance, a commonly used gait speed test is the so-called “four-meter gait speed test”. In this test, the time spent by the patient to walk at their normal pace for four meters is measured [11,12]. 

Recently, smart and pervasive environments, especially in the healthcare field, have been frequently discussed. The technologies behind this Internet revolution, the so-called “Internet of Things (IoT)”, are wearable devices, embedded systems, mobile apps, and cloud and Bluetooth Low Energy (BLE) technologies. Certainly, the use of IoT-enabling hardware and software technologies will play a crucial role in the creation of innovative and unobtrusive Ambient Assisted Living (AAL) systems that can offer useful support for early diagnosis and monitoring of patients affected by sarcopenia. Actually, there are many wearable devices that evaluate human motion, human body balance, distance, physical activity, and physical activity intensity. Many studies have shown that these devices can slow the functional decline of elderly people [13,14,15,16,17].

In this work, enabling IoT hardware and software technologies has been combined in order to create a complete system for detecting all the three sarcopenia-related parameters using a unique device. Moreover, the realized system tries to satisfy very important constraints such as low-power, low-cost, and portability at domestic level. To our knowledge, this is the first wearable device for evaluating all the three essential diagnostic components of sarcopenia to help in the diagnosis and monitoring of this condition over time.

The aim of this work is to realize a wearable device capable of detecting and monitoring sarcopenia, permitting us to facilitate its diagnosis and monitoring over the time, improve the adverse outcomes related to it, and realize tailored activity programs to treat it. In addition, the possibility to collect a lot of data about every person while respecting the relative privacy and ethical rules can enhance our knowledge about this disease and how to cure it.

Other advantages of the application of this solution could be the possibility to avoid exposure for the patients to ionizing radiation, to adopt it in different settings (residences, hospitals, or nursing homes), to increase compliance to specific treatments, and to make early diagnoses.

The proposed system has been included in a first validation in a research laboratory aimed to demonstrate the sensors performance and the system effectiveness.

The paper is structured as follows. In Section 2, similar solutions for the detection of sarcopenia-related parameters are summarized. In Section 3, the main system requirements are analyzed, whereas in Section 4, the materials and methods used to realize the proposed solution are presented. The proposed software architecture is described in Section 5, with details about each system component. The testing procedures to validate the whole system from a functional and performance point of view are presented in Section 6. In Section 7, a discussion on results is reported, as well as a comparison of the proposed solution with similar ones reported in the state of the art. Finally, conclusions are drawn in Section 8. 

## 2. State of the Art

In the literature, there are several works dealing with the use of devices for the detection of the muscular state of a person. Most of these works aim to detect the muscle mass, muscle strength, and/or physical parameters for several purposes and with several sensing technologies. In most cases, commercial sensors are used, and only a few studies propose ad-hoc designed sensors. In addition, some solutions exploit wireless technologies to transfer data to external applications able to gather and process detected data. In particular, there are several works dealing with the use of Electromyography (EMG) sensors to retrieve information about the muscle mass. 

In reference [18], a prototypal wearable device that embeds both EMG sensors and galvanic skin response (GSR) sensors is used to detect driver fatigue symptoms. The sensed EMG and GSR signals are transmitted to a mobile device through BLE technology. A fatigue monitoring application installed in the mobile device is able to detect the driver vigilance level. If the application indicates that the driver vigilance level has dropped to dangerous pre-defined threshold, a vibration warning is triggered to alert the driver. In reference [19], the authors propose a body sensor network with electromyogram and inertial sensors (i.e., accelerometers) to evaluate muscular activities and assess human balance for the purposes of rehabilitation, sports medicine, gait analysis, fall detection, and diagnosis of many diseases associated with a reduction in balance ability. This solution consists of two subsystems operating in parallel—an inertial sensor subsystem and an EMG sensor subsystem. The inertial sensor subsystem is a body sensor network of two nodes. One node is placed on the body of the subject, and the other is connected to a desktop computer. Accelerometer values are transmitted to the node connected to the computer by the node on the body. The EMG sensor subsystem is a commercial wearable signal acquisition system. Data are digitized and wirelessly transmitted to a receiving host computer where the signals are recreated and accessible in analog form. 

An ad-hoc sensor is proposed in reference [20], where a new low-power and low-cost ECG and EMG sensor for biometric and medical applications is presented. Its small size makes it usable in wearable devices. Moreover, experimental tests demonstrated its high sensitivity and its ability to capture even small muscle movements. In reference [21], the realization of a novel EMG sensor for general-purpose IoT applications is reported. The proposed sensor has been validated and compared with commercial EMG sensors, proving it to have better performance in terms of signal-to-noise ratio (SNR).

A flexible wristband for EMG gesture recognition, designed on a flexible printed circuit board (PCB) strip and powered by a small form-factor flexible solar energy panel, in presented in reference [22]. The authors paid close attention to the energy consumption of the device, which is capable of achieving more than 500 h with a single 200 mAh battery and perpetual work with a small form-factor flexible solar panel. In the actual version, the device is able to recognize five different hand gestures. It is not able to communicate with an external application to visualize the processing results, but the outputs are displayed using five different LEDs placed on the board, although, the data (classification and raw data) can also be streamed via the UART ports of the MCU for debugging purposes. 

In reference [23], a prototype of a new smart sock able to detect EMG signals coming from the Gastrocnemius-Tibialis muscles of the leg was realized. Such muscles appear relevant for the assessment of age-related changes in gait, sarcopenia pathology, postural anomalies, fall risk, etc. The hardware was realized by customizing commercial devices for that purpose. The device is mainly made of hybrid polymer electrolyte (HPe) electrodes to contact the skin, an electronic interface unit to read the signals coming from the electrodes, and an elaboration and wireless transmission unit. In particular, the developed sock can send data through a BLE connection. The same authors [24] propose a wearable system able to evaluate real-time risk of fall in elderly people. This system is also based on commercial EMG sensors and ensures wireless data transmission to a real-time application in a range of about 20 m in free space. 

A solution to detect human gait is proposed in reference [25], where authors use a motion sensor and a serial/Bluetooth converter module along with some other components to realize a pre-fall detection system. All sensors are attached on a belt-like structure, which must be worn near the waist area. Moreover, an Android application was developed to receive data from the pre-fall detection system using BLE.

None of the analyzed solutions allows us to fully achieve the intended purpose to provide a unique and portable at domestic-level tool for the detection and monitoring of sarcopenia in a patient. However, each solution allows us to detect some of the parameters of interest for different purposes. Probably, only the solutions proposed in references [19] and [23] could be useful in the diagnosis of sarcopenia, but the only sarcopenia-related parameter that they are able to detect is the muscle mass. Furthermore, these works do not offer cut-off values useful for determining whether a patient is affected by sarcopenia or not. 

## 3. System Requirements

The overall system, which is the subject of the presented study, must necessarily be composed of a hardware component capable of detecting all sarcopenia-related parameters and a software component capable of processing the data coming from the hardware component, storing them, and making them available to the end user. In particular, the software sub-system must be designed in order to guarantee a high degree of decoupling among the various components, which will result in the greater robustness, modularity, and maintainability of the software.

The hardware component has to be realized by exploiting low cost and low power IoT-enabling technologies and must be non-invasive for patients and easy to use. It must be portable to be used directly by the patients in their homes and respecting a simple clinical protocol. In particular, it has to consist in a wearable device able to integrate a set of sensors for retrieving muscle mass, muscle strength, and patient gait speed. It must be equipped with an autonomous power source (i.e., a battery). The use of devices designed for rapid prototyping and for subsequent large-scale and low-cost industrialization should be guaranteed. Moreover, the hardware component must be decoupled from the software component that stores and processes patient’s data. 

The software component should adopt appropriate mechanisms in order to guarantee security in data transmission to the cloud. Furthermore, the presence of Representational State Transfer (REST) Application Programming Interface (API) must be guaranteed in order to facilitate integration with third party systems. A quick and simple access to the data collected for each patient must be guaranteed by using any device (i.e., pc, smartphone, tablet, etc.) through a web application. The web application should allow visualization of all data related to the monitored patients, including all past measurements, in order to monitor the progress of the disease. The system meant to collect data coming from the hardware device must be based on a mobile application, privileging the Android platform. The logic that deals with pre-processing sensory data and calibrating sensors must reside on the mobile application so that it can be updated remotely (through the release of application updates).

The system must not require the presence of any fixed network structure or assume the presence of a local server, but the data must be stored and processed in the cloud. The collected data must be exported anonymously as open data. 

## 4. Materials and Methods

The overall system architecture is based on a RESTful approach in order to guarantee a strong decoupling among the following three subsystems: the embedded system, the mobile app, and the back-end application in the cloud. 

**Figure 1:**
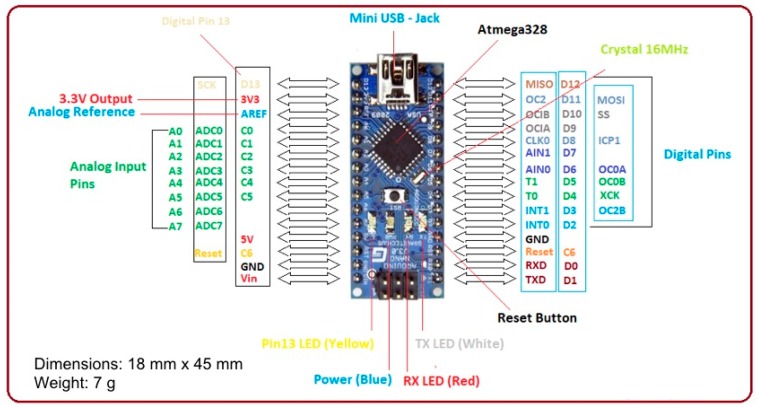
Arduino nano board.

The proposed prototype wearable device is based on an Arduino Nano, Monza (MB), Italy [26] (Figure 1) equipped with an ATMega 328P microcontroller by Microchip Technology Inc., Arizona, USA, powered at 5 V, with a 32 KB flash memory and a 2 KB SRAM. The board provides 30 output pins (eight analogue pins and 22 digital pins) and is able to guarantee a power consumption of only 19 mA. Through the internal regulators it is possible to power the board from 7 V to 12 V, enabling the use of a single 700 mAh 9 V battery. This battery guarantees an operating life of about nine hours. It has been placed in the in the board case in order to be easily replaced in case of dead battery.

**Figure 2:**
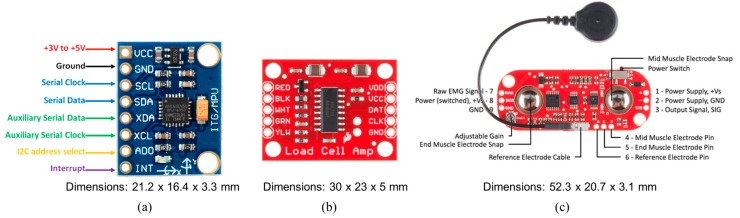
GY-521 board (**a**), HX711 board (**b**), and MyoWare board (**c**).

To meet the project requirements, small and low-cost sensors compatible with the microcontroller were selected. The GY-521 board, San Jose, CA, USA, shown in Figure 2a, was used to detect patient gait speed. It embeds the InvenSense MPU-6050 chip, San Jose, CA, USA, [27] that contains a three-axis Micro Electro-Mechanical Systems (MEMS) accelerometer and a three-axis MEMS gyroscope. In particular, the gyroscope measures the angular acceleration of a body on its own axis, while the accelerometer measures the acceleration of a body along a direction. It is very precise, as it has a 16-bit analog-to-digital (AD) converter for each channel. Therefore, it captures the x, y, and z channels simultaneously. The sensor uses the I^2^C standard communication protocol, which makes it easy to interface with the Arduino boards, Monza (MB), Italy. In order to measure the muscle strength, the HX711 board by SparkFun Electronics, Niwot, CO, USA, [28], consisting of a load cell and a circuit to amplify the signal has been selected (Figure 2b). Its operating principle is based on deflection. Based on this principle, when force is exerted on the load cell, it produces an alteration in the value of the resistance placed inside the sensor. The resulting alteration can be measured as voltage. The change in voltage is proportional to the amount of force applied to the cell, thus the amount of force can be calculated from the load cell’s output. 

Indication about the muscle mass are obtained through an EMG sensor connected to the Arduino Nano board, that is the MyoWare Muscle Sensor board by Advancer Technologies, Raleigh, NC, USA (Figure 2c) [29,30,31]. The MyoWare Muscle Sensor measures, filters, rectifies, and amplifies the electrical activity of a muscle and produces an analog output signal that can easily be read by the microcontroller.

To develop the mobile application dedicated to gather and process all data by sensors and to associate the processed data with the patient’s personal data, the operating system Android by Google LLC, California, CA, USA, was selected. In particular, the mobile application was developed using Android Studio IDE, widely used and appreciated by many developers for its robustness and simplicity.

Spring Boot by Pivotal, San Francisco, CA, USA [32] was used to realize the back-end web service. Spring Boot makes it easy to create stand-alone, production-grade Spring based Applications that you can “just run.” Among its main features, it provides “starter” dependencies to simplify your build configuration and it automatically configures Spring and third-party libraries whenever possible. To store acquired data, a PostgreSQL Database Management System (DBMS), California, CA, USA, [33] was used. PostgreSQL is one of the major relational database management systems that are well received in the world of application development and is also the most advanced SQL-compliant and open-source objective Relational Database Management System (RDBMS). 

As the last technological choice, the Angular framework by Google LLC, California, CA, USA [34] was used to develop the Web application that acts as front-end of the whole system. It is a very popular and widely used JavaScript framework for building mobile and desktop web applications.

## 5. Proposed System Architecture

**Figure 3:**
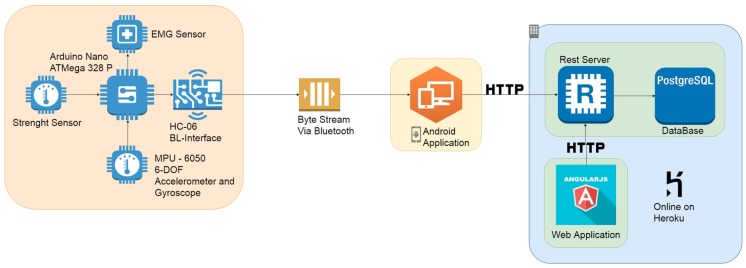
Proposed system architecture.

The proposed system architecture is composed of the following four sub-systems: (i) the embedded and wearable system, (ii) the mobile application, (iii) the REST server connected with a PostgreSQL database, and (iv) the web application (Figure 3).

The embedded system is in charge of data detecting and gathering by several connected sensors, performing some simple processing and sending the information to the mobile application via Bluetooth. To meet the system requirements, the following three small, low-cost sensors were connected to the electronic board:

- the GY-521 board embedding the InvenSense MPU-6050 chip used to calculate the patient gait speed;
- the HX711 board as force sensor;
- the MyoWare board measuring muscle parameters used to calculate muscle tone and activity.

Once connected to the prototypal wearable device, the sensors were calibrated by setting both offset and full-scale values in order to ensure more reliable measurements.

The mobile application allows us to perform the following operations: (i) insert patient data, (ii) gather sensors data from the wearable device, (iii) pre-process received data in order to extrapolate significative information, and (iv) format the processed data in a JSON file and send them to the server for storage. To store acquired data, a PostgreSQL DBMS was used, managed by a RESTful web service that also provides REST APIs useful to manage stored data. Finally, the Web application acts as front-end of the whole system. Developed in Angular, it allows us to query the database through the RESTful web service and to display both the personal data and health data of the patient.

### 5.1. Embedded Sub-System

**Figure 4:**
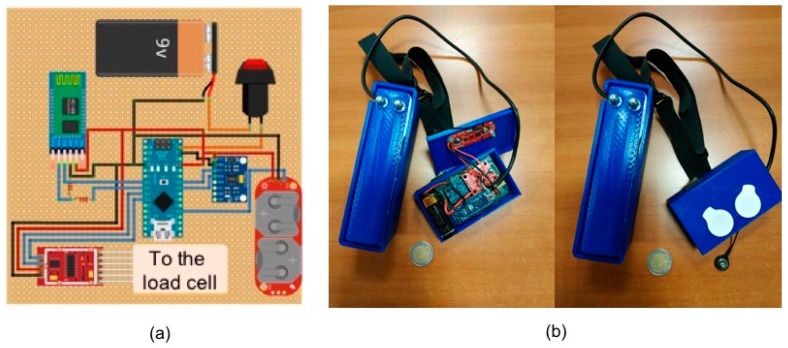
Prototypal wearable device. Design of the electronic hardware (**a**) and realized device (**b**).

In Figure 4a, the design of the electronic hardware to acquire parameters related to sarcopenia is reported, whereas Figure 4b shows a photo of the realized prototypal device.

**Figure 5:**
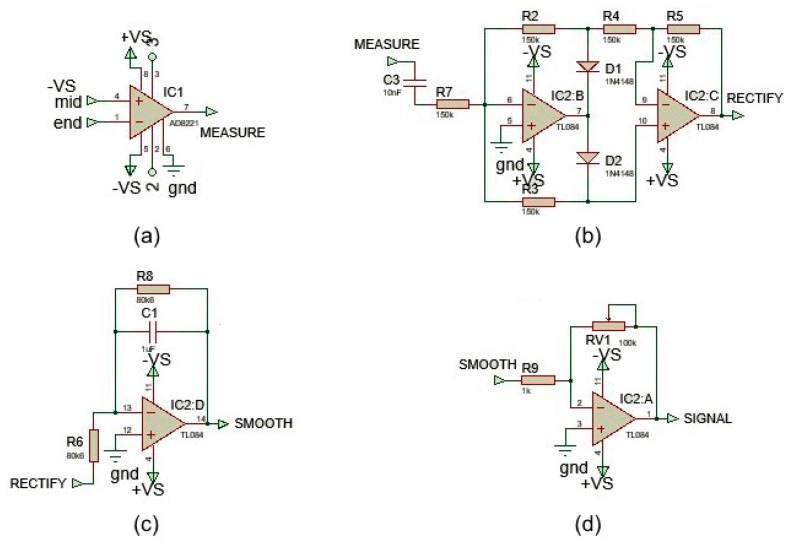
Signal conditioning steps. Difference between first and second electromyographic signals (**a**), signal rectification (**b**), signal smoothing (**c**), and variable signal amplification (**d**).

In order to acquire the EMG signal, a three-electrode configuration (two differential input and a ground reference) was used. Since the EMG voltage range detected by electrodes is very low and the signal is very noisy due to external sources (such as the power coupling at 50 or 60 Hz), it is necessary to amplify and filter the EMG signal appropriately using an analog-to-digital converter (ADC). In order to carry out these signal conditioning operations, an electronic board produced by SparkFun Electronics, Niwot, CO, USA, the MyoWare Muscle Sensor v3 [35], was used. The board includes several active and passive electronic components that allow the EMG signal conditioning so it can be acquired by the ADC of the microcontroller. The circuit is mainly composed of operational amplifiers and passive components (e.g., resistors, capacitors and diodes) and all the operational amplifiers are powered with a dual power supply. The signal conditioning includes the following operations: (i) the calculation of the difference between first and second electromyographic signals (Figure 5a), (ii) signal rectification (Figure 5b), (iii) signal smoothing (Figure 5c), and (iv) variable signal amplification (Figure 5d).

The first step was realized through a low cost, wide supply range instrumentation amplifier, with rail-to-rail output—the AD8226 by Analog Devices Inc., Norwood, MA, USA [36]. This operational amplifier (OP-AMP) calculates the difference between the signals V+ and V- and amplifies the difference of a factor K which depends on the resistance present between pins 2 and 3. In this case, a resistor of 240 Ω guarantees a signal amplification of about 200.

The second step consists of rectifying the output signal from the OP-AMP. Before the rectification circuit, a capacity was inserted in order to couple the AC signal and remove the DC components. Specifically, by using a capacity of 0.01 μF, spectral components below 106 Hz were suppressed. Then, the obtained signal was rectified by a diode network and two Junction gate Field-Effect Transistor (JFET)-input operational amplifiers (TL084 by Texas Instruments, Dallas, TX, USA [37]). In this step, the negative signal was reversed and transformed into a positive signal (full-wave rectification), thus allowing the calculation of the EMG signal power.

The third step is useful to calculate (with a good approximation) the amplitude envelope shape of the signal in order to give an effective indication of the EMG signal power. This is implemented thanks to an active first-order low-pass filter, using the same TL084 OP-AMP. The low pass filter has a cutoff frequency at about 2 Hz, and therefore, the signal is smoothed by removing the high-frequency spectral components. 

Finally, the last step is necessary to further amplify the smoothed signal to adapt it to the full-scale value input range of the ADC. This operation is performed by another TL084 OP-AMP configured as an inverting amplifier. The amplification level is set by the potentiometer located on the OP-AMP feedback network. The signal resulting from this last stage is read by the ADC. Signal acquired by microcontroller are sent to the dedicated mobile app via Bluetooth through a serial/Bluetooth converter, the HC-06 device by Olimex Ltd., Plovdiv, Bulgaria [38], able to receive data from the microcontroller via a serial port and to convert this data into a Bluetooth signal. 

**Figure 6:**
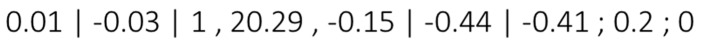
Example of data stream sent by the wearable device to the mobile application.

An example of data stream sent by the wearable device to the mobile application is reported in Figure 6. 

Each value in this data stream is related to a specific sensor based as reported in Table 1. 

Once the data stream is received, the mobile application will be able to extract the substring corresponding to a specific sensor based on the schema in Table 2. 

This components choice allowed to contain the total cost for a single prototypal device within 100€. The main unit is about 55 mm × 94 mm × 7 mm, whereas the load cell unit is about 40 mm × 146 mm × 60 mm, allowing us to satisfy the portability constraint. However, in this work, only a first prototype is presented. The industrialization phase will allow us to further reduce cost and dimensions of the wearable device.

### 5.2. Back-End Server

The back-end server is in charge of storing and processing all information coming from the mobile application. Moreover, it exposes public APIs that can be used to retrieve stored data. Specifically, it implements the REST paradigm, allowing GET/PUT/POST/DELETE HTTP requests. The RESTful APIs are reachable via URL endpoints. In particular, for the interaction between mobile application and server, the following POST APIs were created:

- */AddPatient*: to add a new patient;
- */AddExam*: to add the results of a test performed on a patient.

Once a request is received, the server performs a check for any duplicate using as primary key the patient’s tax code for the first request and the timestamp for the second one. 

For the interaction between the front-end and the server, the following GET APIs were created:

- */getPatients*: to display the list of all patients;
- */getById*: to extract all data relating to the patient corresponding to a specific ID (i.e., the tax code);
- */getByName*: to extract all data relating to the patient corresponding to a specific name;
- */getExams*: to obtain all exams associated to a patient;
- */getExam*: to obtain exam details.

**Figure 7:**
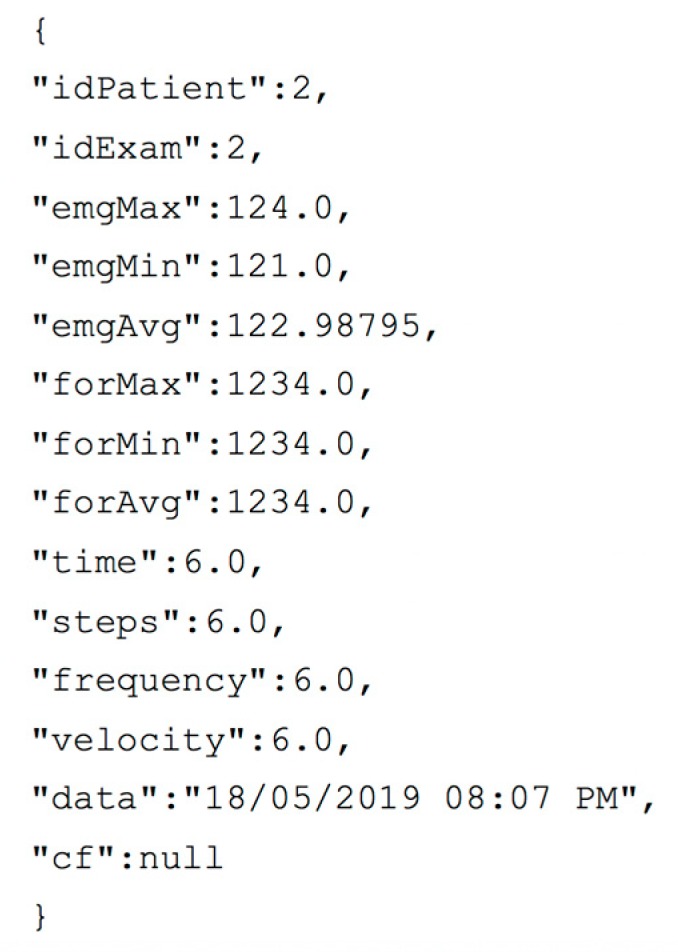
Example of JSON file sent by the mobile application in/*AddExam* API request.

Data interchange between mobile application and back-end server uses the JavaScript Object Notation (JSON) data format. In Figure 7, a JSON file related to the/*AddExam* API is reported as an example of this interaction.

Currently, the back-end server has the simple role of storing data and making them available when needed. However, the introduction of a business logic module is foreseen in order to provide more elaborate information useful to predict the evolution of the disease or to provide suggestions about the actions to be taken to delay its effects.

### 5.3. Mobile App and Front-End Application

The developed mobile App is able to guarantee the following five tasks: (i) insert the patient’s personal data, (ii) connect the app with the prototype wearable device via Bluetooth, (iii) acquire the sensors’ data flow, (iv) process data in order to obtain reliable information on single parameters, and (v) check acquired data and send them to the server.

In the first phase, the medical operator enters the patient’s personal data manually or by scanning the patient’s tax code electronic card. Then, pressing a dedicated button, the operator connects the app with the device worn by the patient in order to acquire sensor data. Then, the operator can choose the exam to perform and proceed to obtain the related measurement. Once the test procedure on the patient is performed, the medical operator obtains a report on received data that can be sent to the server. Alternatively, the operator can choose to repeat the exam or proceed with a different exam and send all the collected data later. Once the exams related to the patient are completed, the mobile application will format all captured data in a JSON file and send it to the server.

The web application communicates with the REST server through the endpoints previously described, which allows the user to search for a patient in order to display both personal and health acquired data.

When the user selects a specific exam related to a patient, he or she can visualize detailed report about test results.

## 6. Experiments

### 6.1. Test Settings

The objective of the tests carried out in this work was to provide a first functional validation of the proposed system in a laboratory environment through a multidisciplinary support approach. This validation served to demonstrate the effectiveness of the proposed system and to evaluate the performance of the sensors used to measure the three considered sarcopenia-related parameters. The performance of the sensors was evaluated by performing several measurements for each sensor (repeated measures technique). Furthermore, these were repeated using two different prototype devices in order to compare the obtained results. To run tests, the following protocol was defined with the support of the medical component of the work group:

(1) Different fixed weights (0.5 kg, 2 kg, 3 kg, and 6 kg) are placed on the load cell to verify its accuracy in detecting the applied load;
(2) The person performs the EMG exam with three electrodes positioned on the superficial extensors of the forearm;
(3) The person walks for 10 m wearing the proposed device.

### 6.2. Proof-of-Concept

**Figure 8:**
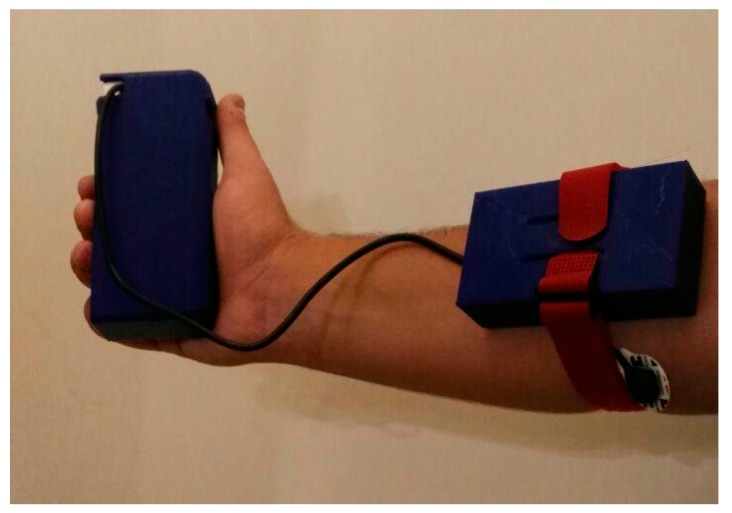
A patient wearing the proposed prototypal device.

In order to validate the proposed system from a functional point of view, a typical scenario was reproduced in the laboratory. The scenario simulates a patient wearing the prototypal device for measuring Sarcopenia-related parameters (Figure 8). In the same scenario, an operator uses the realized mobile application to capture these parameters and the web application to consult them later.

It is important to note that the realized wearable device is not intended for real-time monitoring—it must be worn only to perform scheduled checks (e.g., once every 15 days). The device is easily wearable, so the patient can wear it independently. However, some indications on how to position it correctly on the forearm can be provided to the patient together with the device (e.g., through a simple user manual).

**Figure 9:**
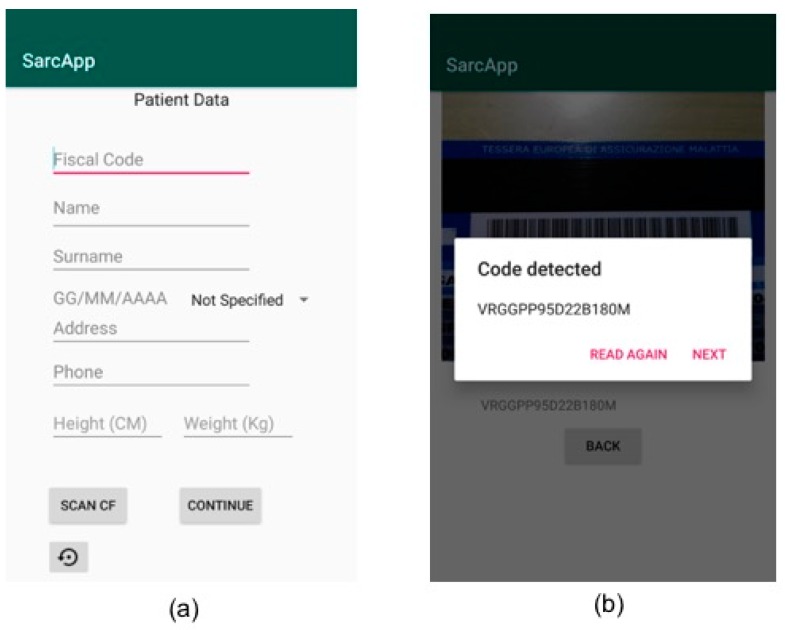
Mobile app screens for the patient data entry. Manual data entry (**a**) and automatic data entry through the scanning of the patient’s tax code (**b**).

As first step, the operator starts the App and inserts the patient’s personal data by filling the related fields that will appear on the mobile device screen (Figure 9a). Alternatively, the operator can scan the patient’s tax code so that some fields are filled automatically by the application (Figure 9b). 

**Figure 10:**
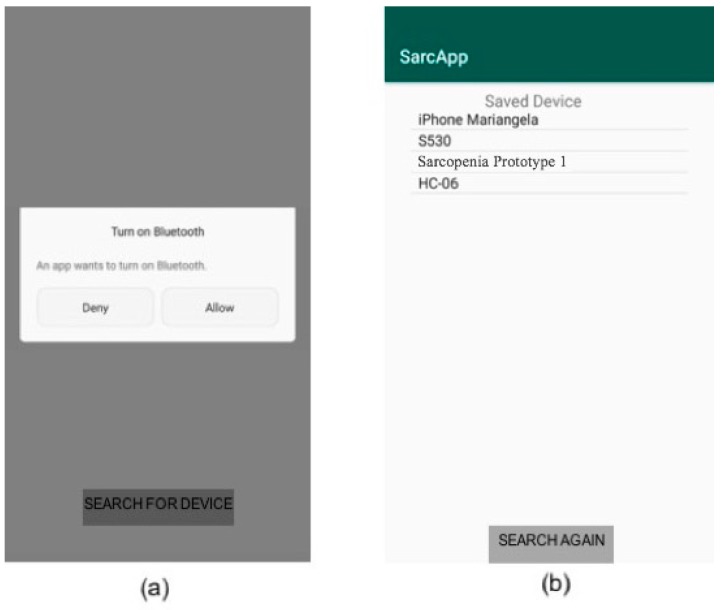
Connection with the wearable device. Bluetooth activation request (**a**) and list of the scanned Bluetooth devices (**b**).

Then, the application asks the consent for the Bluetooth activation on the mobile device, if it is not active (Figure 10a). Through the “scan device” button, the application scans for nearby Bluetooth devices. Then, the operator can select the correct medical device from the list that appears on the screen (Figure 10b).

**Figure 11:**
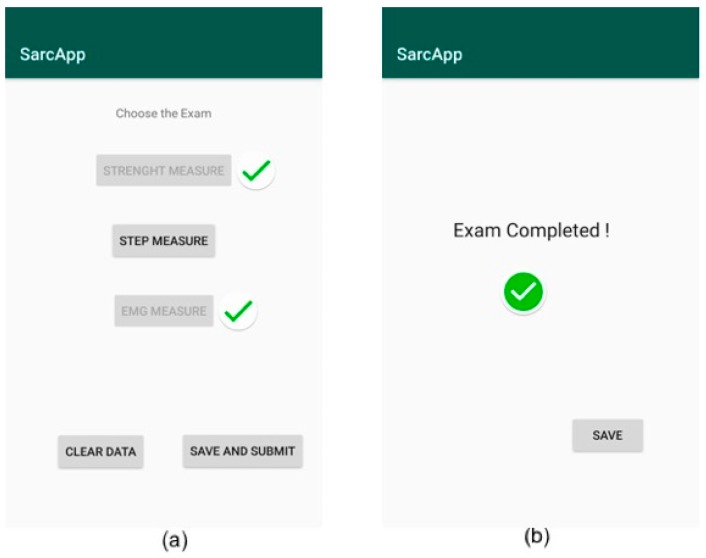
Gathering of the sarcopenia-related parameters. List of monitored/non-monitored parameters (**a**), indication about a completed exam with possibility to locally save data (**b**).

In the next screen, the operator chooses to perform strength measure, step measure, or EMG measure and starts collecting data (Figure 11a). Then, the operator locally saves the captured data and chooses to perform the measurement related to another parameter (Figure 11b). Finally, the operator can send all collected information to the server by pressing the “save and submit” button in Figure 11a.

**Figure 12:**
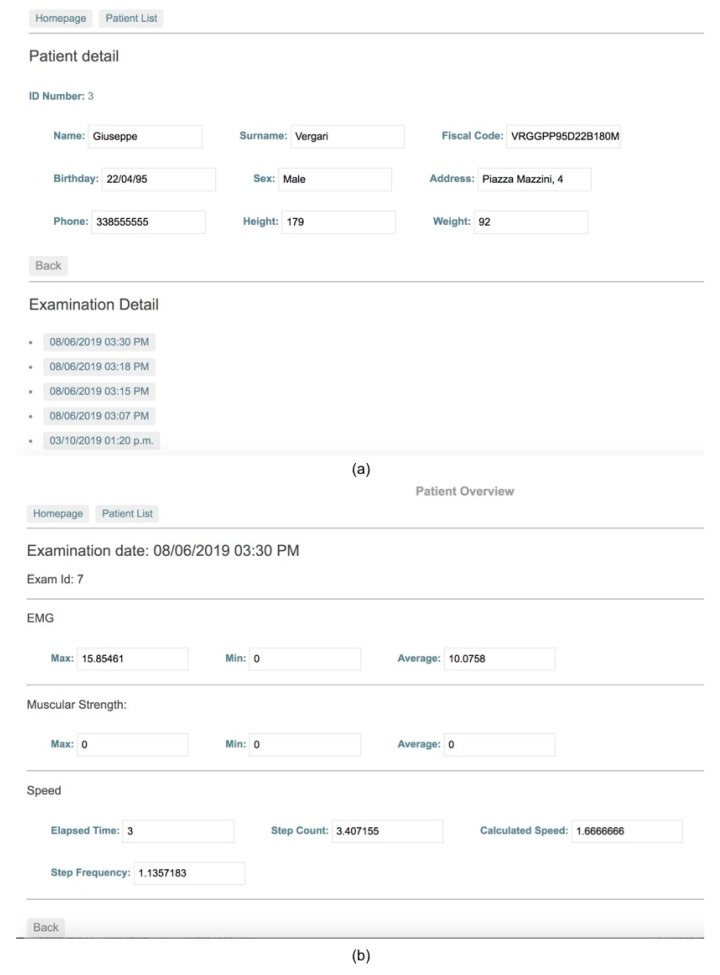
Web application for patient’s data consultation. List of patient’s exams (**a**) and exam details (**b**).

As final step, the operator accesses the web application to check the medical exam performed on the patient and compares them with the values of previous exams, if available (Figure 12a,b). 

### 6.3. Performance Validation

As explained in the test setting section, several tests were performed in order to validate the performance of the sensors selected in the realization of the prototypal device.

All results reported in this paper are characterized by a confidence level equal to 95% with maximum relative error of 5% calculated by applying the very well-known independent replication method. This means that each test has been performed considering a number of replications large enough to guarantee the desired statistical constraints. In particular, for each test, the statistical constraints have been obtained by averaging data collected during no more than 10 replications.

To evaluate the load cell performance, five different loads were considered—0.5 kg, 2 kg, 3 kg, and 6 kg. For each load, five different measures were performed, and same measures were repeated with two different prototypal devices. Results are reported in Table 2.

To evaluate the EMG sensor performance, the three electrodes were positioned on the superficial extensors of the forearm of a person. In Table 3, measures were reported both for the person in a resting position and when muscle is contracted. It is important to highlight that the conventional EMG value for the considered muscle is 18 ± 8 µV.

Finally, to evaluate the performance of the accelerometer and of the related step counter algorithm, the person has walked for 10 m and value obtained by the step counter algorithm were reported in Table 4.

The results obtained for the load cell show sensor precision and accuracy. With regard to the EMG sensor and the accelerometer, the sensors produced precise results, but a small calibration is still necessary in order to improve the accuracy of the measurements. However, the results obtained in this phase are very useful to pave the way for the experimentation on real patients and for a comparison with other instruments currently used for the detection of sarcopenia-related parameters.

## 7. Discussion

In Table 5, a comparison between the proposed solution and all solutions analyzed in Section 2 is presented, which underlines the strengths and weaknesses of each of them. For the comparison, some fundamental system requirements that characterize a system for monitoring sarcopenia have been considered.

The comparison shows that there is no a unique device portable at a domestic level, with low cost and low power, capable of measuring all three parameters related to sarcopenia, demonstrating that the proposed solution could represent an innovation in this field. Moreover, another strength of the proposed solution lies in the presence of a software infrastructure for storing, processing, and displaying information. This paves the way for further development of the system also as a predictive tool and not only for the diagnosis and monitoring of sarcopenia.

The major weakness of the proposed solution with respect to some of the solutions in the literature is certainly the fact that the proposed device is not a certified medical device yet. However, as repeatedly stated, the device is still in a prototype version, but the first steps to start the process of certification and marketing of the device are taking place. First, an intense experimentation will be carried out in order to establish cutoff values that allow us to classify the examined cases as positive or negative to sarcopenia. In particular, regarding muscle strength and walking speed, the proposed device uses techniques similar to those currently used in the medical field to detect the same parameters (i.e., hand grip and accelerometer), and the performed tests will only be used to correctly calibrate the prototypal device. The real challenge resides in the use of the EMG sensor for the detection of muscle mass. Currently, there are cutoff values about skeletal muscle mass for the diagnosis of sarcopenia relating to DXA and BIA, but not relating to EMG. Our challenge, through the subsequent test phase with real patients, is to establish a correlation between the values of muscle mass detected with traditional methods (i.e., DXA and BIA) and those detected with the EMG sensor in order to establish valid cutoff values for sarcopenia diagnosis. After this experimentation, the device will be subject to all the necessary checks to obtain certification (e.g., electromagnetic compatibility tests, electrical safety checks, etc.).

Unlike other similar systems, the wearable device is not autonomous in transmitting information to the server—the mobile application is necessary to act as a gateway. This allows for a simple update of the software component dedicated to data processing (through the release of an updated version of the same application), but it does not guarantee a total decoupling between the hardware component and the software component of the system. To make this possible, the wearable device should have a dedicated communication interface, such as a Wi-Fi or 4G connection.

The results obtained in the performed tests will allow us to refine the proposed system for experimentation with a cohort sample of patients affected and not affected by sarcopenia with the aim of validating it through a comparison with the results obtained with the methods and tools currently adopted by the scientific community. This would allow us to refine the clinical protocol to be adopted and to validate it with real measurements of the single parameters. In this regard, with the collaboration of the medical component of the work group, the following first protocol was defined to be used in the experimentation with real patients:

(1) The patient will be invited to grip the bar connected to the strength sensor with the main hand as hard as possible. After 10 min, the measurement will be repeated with a standard hand grip dynamometer approved as a medical device;
(2) The patient will perform the four-meter walking speed wearing the proposed device. Consequently, we will have the measurement in seconds of the test and the data from the device expressed in meters per second;
(3) The patient will perform the BIA exam with four electrodes using an approved medical device.

## 8. Conclusions

In this work, the challenge of creating a wearable device able to detect the three main parameters related to sarcopenia was addressed. In particular, a complete hardware and software system capable of achieving this goal was designed and implemented. Furthermore, the proposed system, unlike the solutions currently adopted in medical clinics, avoids the exposure of the patients to ionizing radiation.

The implementation of this system could lead to a slowing in of progression of the disease and a reduction of the functional declining, with a possible effect on the mortality, as shown in some interesting studies [39]. An additional benefit could be to reduce the associated health cost related to this condition, considering that the average health care costs for those with mobility disability are 10-fold higher than for those without [40].

The system has been validated in the laboratory both from a functional and performance point of view. The current prototype has been designed to validate the proposed solution, but a reduction in costs, size, and power consumption will be reached in an industrialized version of the system. As ongoing work, a validation of the system on real patients and a comparison with other systems and tools used for the evaluation of the same parameters has begun. Furthermore, a process of industrialization of the wearable device will be undertaken, and a multiplatform application for data collection will be implemented. A possible extension of the system is foreseen by introducing mechanisms for activity recognition. In this way, the device, properly redesigned, could be continuously worn by the patient in order to predict the onset of possible health risks.

## Figures and Tables

**Table 1:** Mapping between data stream values and sensor values.

Accelerometer (g)			Temperature (°C)	Gyroscope (°/s)			EMG (µV)	Strength (kg)
X	Y	Z		X	Y	Z		
0.01	–0.03	1	20.29	–0.15	–0.44	–0.41	0.2	0

**Table 2:** Load cell measures.

		Load [kg]			
		0.5 kg	2 kg	3 kg	6 kg
Prototypal device 1	1	0.51	2.01	2.98	5.95
	2	0.50	2	2.98	5.98
	3	0.51	2.01	2.99	5.96
	4	0.50	2.01	2.99	5.98
	5	0.50	2.01	2.98	5.99
Prototypal device 2	1	0.50	2.01	2.98	5.98
	2	0.50	2.01	2.98	5.98
	3	0.50	2	2.98	5.97
	4	0.51	2	2.98	5.99
	5	0.50	2	2.99	5.99

**Table 3:** Electromyography (EMG) measures.

	EMG Value [µV]	
	Resting Position	Contract Muscle
Prototypal device 1	19.8	346
	25.8	349
	20.8	342
	20.8	334
	22.8	321
Prototypal device 2	19.9	316
	20.6	369
	20.9	312
	21.4	354
	20.4	321

**Table 4:** Value obtained from the step counter algorithm.

	Calculated Steps	Real Steps
Prototypal device 1	6.5	6
	8.2	7
	7.1	6
	6.6	6
	6.8	7
Prototypal device 2	6.9	6
	8.1	7
	5.8	6
	6.2	6
	6.4	7

**Table 5:** Comparison between proposed solution and other solutions existing in the literature.

	Ref.	[18]	[19]	[20]	[21]	[22]	[23]	[24]	[25]	Proposed Solution
Requirement										
Muscle mass (EMG)		√	√	√	√	√	√	√		√
Muscle strength										√
Gait speed (inertial sensors)			√						√	√
Portability		√	√	√	√	√	√	√	√	√
Ability to communicate with external applications		√				√		√	√	√
Low cost		n.a.	n.a.	√	√	n.a.	n.a.	n.a.	n.a.	√
Low power		n.a.	n.a.	√	√	√	n.a.	n.a.	√	√
Certified medical device			√							
Automatic software upgrade		√							√	√
Hardware/software decoupling						√				
